# Correction to articles (as listed below) missing a graphical abstract

**DOI:** 10.1093/esj/aakag034

**Published:** 2026-07-21

This is a correction to the following papers:

Carolijn J M de Bresser, Robbert B M Wiggers, Roos A M van Heeswijk (*et al*), Temporal trends in short- and long-term outcomes after carotid interventions for symptomatic or asymptomatic stenosis: a systematic review and meta-analysis, *European Stroke Journal*, Volume 11, Issue 1, January 2026, aakaf002, https://doi.org/10.1093/esj/aakaf002

Timothée Werlé, Florent Wijanto, Emilien Micard (*et al*), Does the susceptibility vessel sign influence the effectiveness of intravenous thrombolysis before endovascular thrombectomy in acute ischaemic stroke?, *European Stroke Journal*, Volume 11, Issue 1, January 2026, aakaf003, https://doi.org/10.1093/esj/aakaf003

Lisa Kaindl, Michail Giannakakis, Joshua Mbroh (*et al*), Thrombectomy with and without emergent stenting in acute ischemic stroke due to carotid artery dissection, *European Stroke Journal*, Volume 11, Issue 1, January 2026, aakaf004, https://doi.org/10.1093/esj/aakaf004

Alice de Vautibault, Adnan Mujanovic, Valentin Amar (*et al*), Cardiovascular risk factor control in patients with covert brain infarcts in the prospective SILENT cohort study, *European Stroke Journal*, Volume 11, Issue 1, January 2026, aakaf006, https://doi.org/10.1093/esj/aakaf006

Philip S Nash, Simon Fandler-Höfler, Gareth Ambler (*et al*), Associations of chronic kidney disease with recurrent stroke in patients with intracerebral haemorrhage, *European Stroke Journal*, Volume 11, Issue 1, January 2026, aakaf007, https://doi.org/10.1093/esj/aakaf007

Umberto Pensato, Costanza M Rapillo, Federico Mazzacane(*et al*), Non-contrast CT findings suggestive of secondary intracerebral haemorrhage, *European Stroke Journal*, Volume 11, Issue 1, January 2026, aakaf010, https://doi.org/10.1093/esj/aakaf010

Sam J Neilson, Natasha E Fullerton, Sin Yee Foo (*et al*), Are lacunar infarcts associated with a “susceptibility vessel sign”? A 7-tesla magnetic resonance imaging study, *European Stroke Journal*, Volume 11, Issue 1, January 2026, aakaf011, https://doi.org/10.1093/esj/aakaf011

Yoel Schwartzmann, Mirjam R Heldner, Hamza Jubran (*et al*), FRET score: predictors of futile recanalisation following endovascular thrombectomy—a multicentre cohort study from the EVATRISP collaboration, *European Stroke Journal*, Volume 11, Issue 1, January 2026, aakaf013, https://doi.org/10.1093/esj/aakaf013

Mary-Helen Søyland, Arnstein Tveiten, Agnethe Eltoft (*et al*), Survival after wake-up stroke and unknown-onset stroke—a nationwide observational study from the Norwegian Stroke Registry, *European Stroke Journal*, Volume 11, Issue 1, January 2026, aakaf016, https://doi.org/10.1093/esj/aakaf016

Mukul Sharma, Qiang Dong, Teruyuki Hirano (*et al*), OCEANIC-STROKE Investigators, Rationale, design and baseline characteristics of participants in the OCEANIC-STROKE trial of FXIa inhibition for secondary stroke prevention, *European Stroke Journal*, Volume 11, Issue 1, January 2026, aakaf017, https://doi.org/10.1093/esj/aakaf017

João André Sousa, Olalla Pancorbo, Renato Simonetti (*et al*), Influence of spot sign on the association between rapidly achieving blood pressure reduction and intracerebral haemorrhage outcomes, *European Stroke Journal*, Volume 11, Issue 1, January 2026, aakaf024, https://doi.org/10.1093/esj/aakaf024

Hanne Christensen, Francesca Romana Pezzella, Melinda Berg Roaldsen (*et al*), Stroke Action Plan for Europe 2018–2030 (SAP-E): mid-term review and update, *European Stroke Journal*, Volume 11, Issue 1, January 2026, aakaf026, https://doi.org/10.1093/esj/aakaf026

In the original publications, the graphical abstract was erroneously missed for each of the above papers. These have now been added to each paper respectively. The publisher apologises for the error. Addendum: published [remove these brackets and italicised text and change xxx for the date this new notice with addendum added publishes] xxx

**Addendum:** published 21 July 2026

The title of the above notice has been corrected to read:

“Correction to articles (as listed below) missing a graphical abstract” instead of: “Correction to:”.

